# Draft Genome Sequence of Coccoid *Lactobacillus equigenerosi* NRIC 0697^T^ Isolated from the Gastrointestinal Tracts of Healthy Thoroughbreds

**DOI:** 10.1128/genomeA.01679-15

**Published:** 2016-02-04

**Authors:** Hidehiro Toh, Akiyo Nakano, Co Thi Kim Nguyen, Iyo Mimura, Kensuke Arakawa, Kosuke Tashiro, Takefumi Kikusui, Hidetoshi Morita

**Affiliations:** a Medical Institute of Bioregulation, Kyushu University, Fukuoka, Japan; b Department of Microbiology and Infectious Diseases, Nara Medical University, Kashihara, Nara, Japan; c Graduate School of Environmental and Life Science, Okayama University, Okayama, Japan; d Graduate School of Systems Life Sciences, Kyushu University, Fukuoka, Japan; e School of Veterinary Medicine, Azabu University, Sagamihara, Kanagawa, Japan; f College of Education, Hue University, Hue, Vietnam

## Abstract

*Lactobacillus equigenerosi* NRIC 0697^T^ was isolated from the gastrointestinal tracts of healthy thoroughbreds. This strain produced unique spherical or oval cells, which is rare in the genus *Lactobacillus*. Here, we report the draft genome sequence of this strain.

## GENOME ANNOUNCEMENT

*Lactobacillus equigenerosi* forms a subcluster in the *Lactobacillus reuteri* group, being closely related to *L. fermentum*, *L. gastricus*, *L. ingluviei*, and *L. mucosae*, in the phylogenetic tree of the genus *Lactobacillus* (1). We previously isolated *L. equigenerosi* NRIC 0697^T^ from the feces of two actively racing thoroughbred horses (1) and deposited strain NRIC 0697^T^ as JCM 14505^T^ and DSM 18793^T^ in the Japan Collection of Microorganisms (JCM) and the Deutsche Sammlung von Mikroorganismen und Zellkulturen GmbH (DSMZ), respectively. This strain forms coccoid cells but not rod-shaped cells that are found in most lactobacilli (1). *L. equigenerosi* is considered to be specific to equines (2, 3) and has high autoaggregative properties. *L. equigenerosi* may be able to be used as a probiotic for equines, because this species is nonpathogenic and does not elicit an allergic response (3).

The *L. equigenerosi* NRIC 0697^T^ genome underwent paired-end sequencing using the Illumina MiSeq platform. Genomic libraries containing 600- to 1,000-bp inserts were constructed and sequenced, yielding 3,584,696 sequences that provided 495-fold coverage from both ends of the genomic clones. The sequence reads were assembled using Newbler version 2.8 (Roche), and the assembled genome consisted of 84 contigs, with a total length of 1,594,458 bp. The genome size was relatively smaller than those of other lactobacilli. The draft genome of *L. equigenerosi* NRIC 0697^T^ contained 1,496 predicted protein-coding genes and 43 tRNA genes. The genome information of this species will be useful for further studies of its physiology, morphology, taxonomy, and ecology.

### Nucleotide sequence accession numbers.

The draft genome sequence for *L. equigenerosi* NRIC 0697^T^ has been deposited in the DDBJ/GenBank/EMBL database under the accession numbers BCMX01000001 to BCMX01000084.
